# The potentially dangerous zone of the dorsomedial cutaneous nerve in minimally invasive surgery for hallux valgus: a cadaveric study

**DOI:** 10.1186/s13018-023-04419-8

**Published:** 2023-12-04

**Authors:** Zhaolin Teng, Xiang Geng, Jiafeng Song, Li Chen, Chao Zhang, Jiazhang Huang, Xu Wang, Xin Ma

**Affiliations:** Department of Orthopedic Surgery, Huashan Hospital, Fudan University, Shanghai, China

**Keywords:** Hallux valgus, Cadaver anatomy, Dorsomedial cutaneous nerve, Minimally invasive surgery, MICA

## Abstract

**Background:**

This study aims to describe the distribution of the dorsomedial cutaneous nerve (DMCN) in the middle and proximal parts of the metatarsal from a lateral view. The purpose is to provide guidance to surgeons in protecting the nerve during the 3rd and 4th generation minimally invasive surgery (MIS) for hallux valgus (HV).

**Methods:**

A total of 20 cadaveric feet were dissected to expose the course of the DMCN and sentinel vein. Measurements of the distances between the nerve/vein and the upper border of the metatarsal, as well as the height of the metatarsal, were taken from a lateral view. The distribution area was then described in proportion.

**Results:**

At the base of the metatarsal, the DMCN was distributed in the upper 25.7% of the area. When it reached the middle of the metatarsal, the DMCN was distributed in the upper 13.2–47.2% of the area. As for the sentinel vein, it was distributed in the upper 23.5–71.9% and upper 4.1–52.7%, respectively, at these two positions.

**Conclusions:**

The area, which is above the line connecting the upper 1/4 point at the base of the first metatarsal and the 1/2 point at the middle of the first metatarsal, is a dangerous zone for the DMCN. Avoiding the zone is recommended during MIS for HV.

## Background

Hallux valgus (HV) is one of the most common forefoot deformities in older individuals, particularly women. It is caused by the degeneration of soft tissues and muscle imbalance [1, 2]. Surgery is typically the primary solution for symptomatic hallux valgus. In recent years, minimally invasive surgery (MIS) has gained popularity due to its esthetic appeal. The 3rd and 4th generation MIS is capable of achieving radiologic and clinical outcomes that are equal to, if not better than, open surgery [3–6]. However, there is a risk of damaging anatomical structures, such as the dorsomedial cutaneous nerves (DMCN), during MIS without an open incision [7, 8]. Several studies have discussed the course of DMCN and methods to prevent nerve injuries at the metatarsophalangeal joint level (MTPJ) during metatarsal osteotomy [9–11]. Normally, screws are inserted from proximomedial to distolateral of the metatarsal, and proximal metatarsal osteotomy can be performed using MIS [12, 13]. As a result, these steps can potentially damage the DMCN at the proximal metatarsal level. The objective of this study is to outline the distribution of DMCN from a lateral view at the middle and proximal metatarsal areas and to identify a safe portal for screw placement and proximal metatarsal osteotomy to minimize nerve injuries.

## Methods

### Specimen dissection

**Fig. 1:**
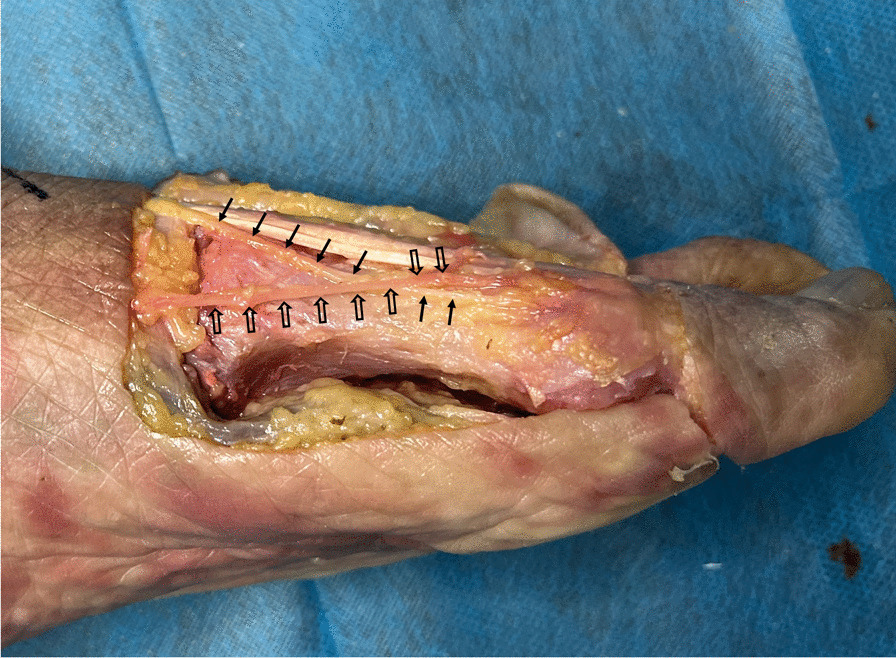
Exposure of DMCN and sentinel vein: Solid arrows show the course of DMCN, and hollow arrows show the course of sentinel vein

This study is an anatomic, descriptive, observational and cross-sectional study. Institutional review board approval was obtained at the beginning of the study. Twenty fresh-frozen cadaver specimens were the subjects of this study. Mild-to-moderate HV deformity was confirmed based on the hallux valgus angle (HVA) measured by foot weight-bearing anteroposterior (AP) X-rays: Mild deformity was defined as 15° ≤ HVA < 20°, while moderate deformity was defined as 20° ≤ HVA ≤ 40° [14]. Intermetatarsal angles (IMA) were also measured by AP X-rays. Using Bernard’s axial projection, pronation angles of the first metatarsal were collected [15, 16]. Donor records indicated no history of foot surgery or injury. Prior to the experiments, the specimens were thawed at room temperature for 24 h. Subsequently, twenty cadaveric feet were dissected to expose the course of DMCN and sentinel vein from a level starting 1-cm proximal to the first tarsometatarsal joint to the metatarsal neck (Fig. 1). The dissection process involved revealing the soft tissue until the first metatarsal bone became visible.

### Course measurement

**Fig. 2:**
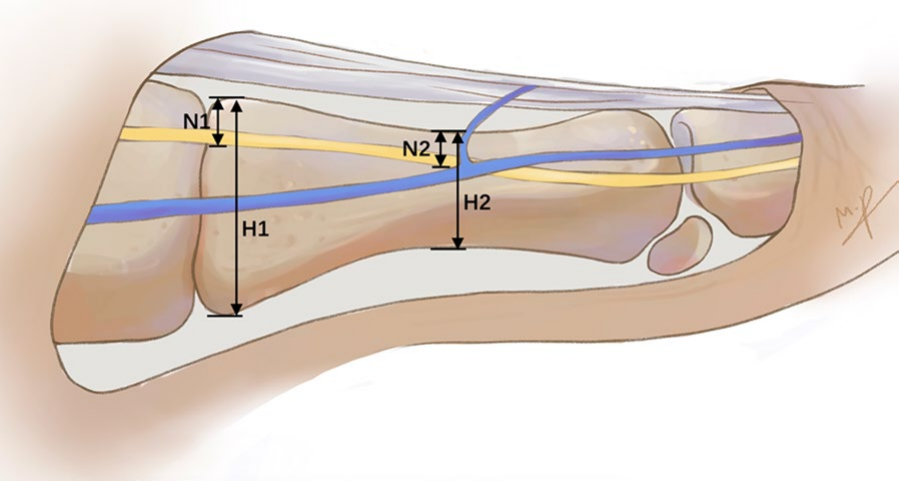
Measurement of the heights of metatarsal and distances between DMCN and upper border of metatarsal

In the lateral view, the height of the first metatarsal base was measured using a vernier caliper and recorded as H1. Additionally, the height of the middle part of the first metatarsal (H2) was measured in the same manner. In this view, the distances from the inferior edge of the DMCN to the uppermost part of the base and middle section of the first metatarsal were recorded as N1 and N2, respectively (Fig. 2). Correspondingly, the distances from the sentinel vein to the uppermost part of the base and middle section of the first metatarsal were recorded as V1 and V2.

### Statistical analysis

The average and standard deviation of the measured widths and distances were calculated. Furthermore, N1/H1 and V1/H1 were calculated and referred to as N1% and V1%, respectively, to describe the distribution of the DMCN and sentinel vein at the level of the metatarsal base. Similarly, N2% and V2% were calculated by dividing N2 and V2 by H2, and these values represented the distribution at the middle section of the first metatarsal bone. Microsoft Excel for Mac 16.72 (Microsoft, Redmond, WA, USA) was used to perform the calculations. Pearson’s correlation analysis was conducted using SPSS (SPSS Inc., USA) to determine the correlation between HVA and the variables mentioned above, as well as the correlation between the pronation angles and variables.

## Results

**Fig. 3:**
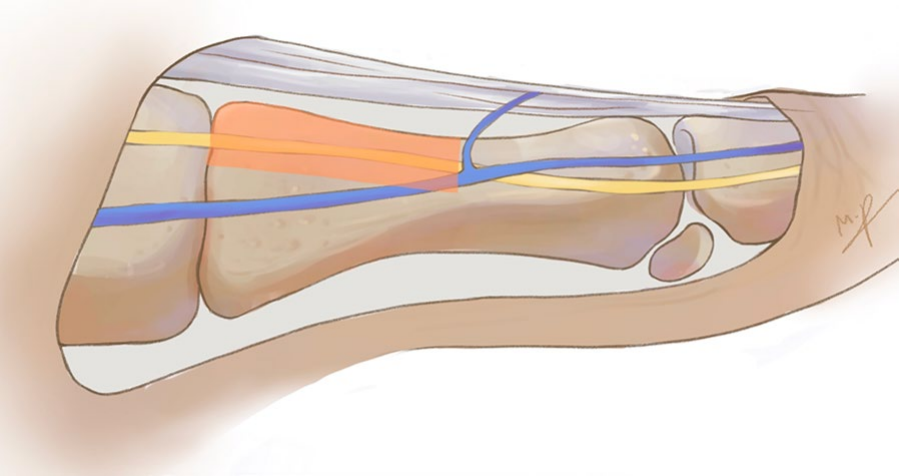
The dangerous zone of DMCN at the proximal half of metatarsal: Red area shows the dangerous zone which is above the line connecting the upper 1/4 point at the base and the 1/2 point at the middle of the first metatarsal

Demographic data regarding age and angles are presented in Table 1. Four donors were male and 16 were female. There were five mild HV deformity and 15 moderate deformity confirmed by X-rays. After dissecting and measuring, we recorded the heights of the metatarsals and the distances of the DMCN and sentinel vein, which are shown in Table 2. The range of N1%, N2%, V1% and V2% is presented in Table 3. At the base of the first metatarsal, the DMCN was distributed in the upper 25.7% area. When the DMCN reached the middle of the metatarsal, its lowest course was close to the midpoint (47.2%) but did not exceed it. In short, we defined the area above the line connecting the upper 1/4 point at the base and the 1/2 point at the middle of the first metatarsal as a dangerous zone for the DMCN (Fig. 3).

**Table 1:** Age and angles of specimens

	Age(y)	HVA (°)	IMA (°)	Pronation angle (°)
Average	64.5 ± 9.1	27.3 ± 7.4	12.6 ± 3.8	14.5 ± 3.9

**Table 2:** Summaries of measured heights and distances

	Average (mm)	Minimum (mm)	Maximum (mm)
H1	21.9 ± 1.6	27.2	33.0
N1	5.5 ± 2.0	0	7.8
V1	13.8 ± 3.5	8.4	21.8
H2	15.1 ± 1.1	12.8	16.8
N2	4.1 ± 1.2	2.0	6.7
V2	4.2 ± 1.5	2.7	7.7

**Table 3:** Range of N1%, N2%, V1% and V2%

	N1%	N2%	V1%	V2%
Range	0~25.7	13.2~47.2	23.5~71.9	4.1~52.7

As for the sentinel vein, its course varied among individuals. The sentinel vein was distributed in the middle part at the base (23.5–71.9%), below the DMCN. At the middle of the first metatarsal, the vein was adjacent to and sometimes even above the DMCN. No significance was found in Pearson’s correlation analysis (*P* > 0.05).

## Discussion

In the surgery for HV, the most commonly damaged nerve is the DMCN. The osteotomy of the first metatarsal is the normal surgical choice. The standard surgical choice involves an osteotomy of the first metatarsal. Research has shown that the incision for the osteotomy is in close proximity to the DMCN at the metatarsophalangeal joint level. It is possible that a “Bunion branch” of the DMCN may exist beneath the incision [11]. In a detailed examination of the DMCN’s course, its relationship with the extensor hallucis longus (EHL) was described. A "Danger Zone" was identified between 12 and 19 mm from the center of the EHL tendon at the level of the MTPJ [9]. These studies provide guidance for surgeons in choosing the osteotomy portal and using an elevator to protect the DMCN in minimally invasive surgery for HV.

Our study focuses on the use of screws in MIS for HV. Although MIS with no fixation or with *K*-wire was reported to be effective, using internal fixation could help patients achieve early weight-bearing while also reduce the risk of superficial infections [17–19]. According to a study on percutaneous fixation in tarsometatarsal fusion with a similar approach to minimally invasive chevron and akin osteotomy (MICA), there were reports of potential irritation and damage to the DMCN [20]. It was suggested that a small incision be made for better visualization of the nerve. In our study, we dissected and described the DMCN at the proximal half of the metatarsal because the screws are typically placed from proximomedial to distolateral in MICA. Most surgeons choose to use two screws parallel to the long axis of the metatarsal in MICA [21]. However, other MIS techniques involving proximal metatarsal osteotomy and different fixation choices can also pose a risk of damaging the DMCN [12]. The area above the line connecting the upper 1/4 point at the base and the 1/2 point at the middle of the first metatarsal, as viewed laterally, is considered the dangerous zone for the DMCN. Therefore, when placing guide pins and screws, it is important to avoid this zone. So far, irritation and injuries of DMCN with screws in MIS techniques are rarely reported, the possible reasons are as follows: (1) Placement of screws parallel to the long axis is the major practice, in which screws are mostly below DMCN or at the borderline of the zone; and (2) postoperative local numbness seems to receive little attention compared to non-union, poor wound healing and recurrence of deformities in many follow-up studies [22]. However, the fact is that numbness actually has a significant impact on patient satisfaction, which is why we conducted this study. Some surgeons may opt to place screws from the dorsal proximomedial to the plantar distolateral direction [23]. While this can provide three-dimensional stability, we recommend against placing the proximal screws dorsally. Avoiding dorsomedial entry not only helps avoid the dangerous zone, but also reduces the risk of plantar placement of the screw, sending the screw more in the center of the head and providing better fixation. In cases involving proximal osteotomy, it is crucial to utilize blunt dissection and protect the soft tissue.

In addition, it has been observed that certain steps in the MIS could potentially cause harm to the DMCN. Following metatarsal osteotomy, some surgeons opt to perform medial eminence resection in order to achieve a more esthetically pleasing outcome. A cadaveric study revealed that in half of the specimens, the DMCN was compromised after undergoing the minimally invasive distal chevron osteotomy and medial eminence resection [7]. However, a separate study found that none of the DMCNs were injured in cases where the MICA was performed without medial eminence resection [24]. Hence, it is recommended to utilize an alternative approach to protect the DMCN when medial eminence resection is deemed necessary.

Lateral soft tissue release plays a crucial role in correcting HV. The distance between the adductor tenotomy portal and the dorsolateral nerve has been reported to be 3.3 ± 1.4 mm [25]. Even in cases of extensive percutaneous lateral release, the dorsolateral nerves remain intact [26]. However, once the release becomes deep enough to transect the deep transverse metatarsal ligament, there is a risk of damage to the plantar nerves [27].

The use of the sentinel vein in surgery for HV has been reported to locate the DMCN [28]. However, in the proximal part of the metatarsal, the courses of the sentinel vein and DMCN are not adjacent. Furthermore, identifying the sentinel vein in MIS can be challenging [29]. Other methods, such as ultrasound, have also been reported for identifying the DMCN, but they are not as simple and convenient in MIS [30]. Based on our study and previous research on the DMCN at the level of the MTPJ, we can effectively protect the DMCN in MIS for HV, thereby preventing post-surgical numbness.

There was no significance in Pearson’s correlation analysis, indicating that the distribution of DMCN was not related with the severity of HV or the pronation of the first metatarsal. Although it has been reported that the distance between DMCN and EHL at the level of the MTPJ is associated with the severity of HV [9], we argue that the displacement of DMCN in the proximal metatarsal is minimal in patients with mild and moderate HV.

Limitation of the study included a small sample size of only 20 specimens, and similar studies with a greater number of samples were suggested. However, the study provides a simple and practical method to avoid DMCN without special tools, which will prevent postoperative numbness. Future, we could evaluate the damage to neurovascular structures by inserting screws into specimens in the 3rd and 4th generation MIS osteotomies.

## Conclusion

DMCN is particularly susceptible to injury in MIS for HV. To ensure safe screw placement and operation of the proximal metatarsal, we recommend avoiding the dangerous zone of DMCN, which is defined as the area located above the line connecting the upper 1/4 point at the base and the 1/2 point at the middle of the first metatarsal.

## Data Availability

Data will be available upon request by the first author.
